# A Selective Review on Information Criteria in Multiple Change Point Detection

**DOI:** 10.3390/e26010050

**Published:** 2024-01-04

**Authors:** Zhanzhongyu Gao, Xun Xiao, Yi-Ping Fang, Jing Rao, Huadong Mo

**Affiliations:** 1 School of Systems and Computing, University of New South Wales, Canberra, ACT 2612, Australia; zhanzhongyu.gao@adfa.edu.au (Z.G.); huadong.mo@unsw.edu.au (H.M.); 2 Department of Mathematics and Statistics, University of Otago, Dunedin 9016, New Zealand; 3 Chair Risk and Resilience of Complex Systems, Laboratoire Génie Industriel, CentraleSupélec, Université Paris-Saclay, 91190 Bures-sur-Yvette, France; yiping.fang@centralesupelec.fr; 4 Key Laboratory of Precision Opto-Mechatronics Technology, School of Instrumentation and Opto-Electronic Engineering, Beihang University, Beijing 100191, China; jingrao@buaa.edu.cn

**Keywords:** Akaike information criterion, Bayesian information criterion, hypothesis test, model selection, piecewise constant, signal processing

## Abstract

Change points indicate significant shifts in the statistical properties in data streams at some time points. Detecting change points efficiently and effectively are essential for us to understand the underlying data-generating mechanism in modern data streams with versatile parameter-varying patterns. However, it becomes a highly challenging problem to locate multiple change points in the noisy data. Although the Bayesian information criterion has been proven to be an effective way of selecting multiple change points in an asymptotical sense, its finite sample performance could be deficient. In this article, we have reviewed a list of information criterion-based methods for multiple change point detection, including Akaike information criterion, Bayesian information criterion, minimum description length, and their variants, with the emphasis on their practical applications. Simulation studies are conducted to investigate the actual performance of different information criteria in detecting multiple change points with possible model mis-specification for the practitioners. A case study on the SCADA signals of wind turbines is conducted to demonstrate the actual change point detection power of different information criteria. Finally, some key challenges in the development and application of multiple change point detection are presented for future research work.

## 1. Introduction

Change points are variations in time series that indicate significant changes in the data’s statistical properties. The accurate identification of change points helps to analyze the underlying state transfer of simple and complex systems. The research on change points has been a longstanding problem in statistics and econometrics since the 1950s and has subsequently found applications in various other fields. Nowadays, this topic has been revitalized as a critical task in numerous domains that rely on signal processing and time series analysis, including image and speech processing, bioinformatics, climate change analysis, and various engineering areas.

The majority of research efforts have been directed towards detecting abrupt or sudden changes in model parameters, with only a few studies considering the assumption of slow and continuous changes in time series. Refs. [1,2] explore the detection of abrupt variance changes within the context of a smooth and continuous mean-shifting trend. To the best of our knowledge, there is a gap in the existing research landscape when detecting abrupt mean changes under a scenario with slow variance shifts and smooth variations in both mean and variance. In this article, we focus on reviewing change point detection involving abrupt variation in time series.

Very early works of change point (CP) detection date back to the 50s in the last century [3,4,5] with discussions on locating a single shift in the mean value of independent and identically distributed (i.i.d.) Gaussian observations. Ref. [6] summarized the detection of a single change point in two cases: (1) the model with two constant means and (2) the model with two intersecting regression lines. The former case has received extensive attention in the studies conducted by [3,4,5], focusing on two constant mean models. On the other hand, the latter case, involving two intersecting regression models, was thoroughly discussed by [7,8] in their respective works on fitting and inference. While there is a rich list of literature related to single CP and while this issue has been thoroughly researched, it is worth noting that assuming only one change point in a signal or time series is often a very restrictive assumption. What is more in line with many real-world scenarios is the state transfer of the signal or time series under consideration may occur multiple times, implying the existence of multiple change points. Thus, multiple change points (MCP) problems have become significant in various fields and have garnered increasing attention more recently.

Based on the nature of data collection, data processing and decision-making, CP methods can be categorized into two main types: (1) *online* CP detection, which aims to identify change points as soon as possible for real-time time series, and (2) *offline* CP detection, sometimes called *retrospective* CP detection or *signal segmentation*, which tries to find out all change points in the historical time series. In the former type of task, under the online detection settings, a trade-off between the false alarm rate and average detection delay needs to be carefully considered. On the other hand, in the latter type of task, the accuracy of detection methods becomes more crucial as there is often no specific time requirement in offline detection scenarios. Generally, MCP detection aims to achieve two objectives. The first objective is to analyze whether there are any change points present in the sequence of observations. The second objective is to determine the optimal number of change points and their corresponding locations [9].

In this article, we will present a selective review of MCP detection methods in offline settings, with a particular emphasis on methods that utilize information criteria. Since the locations and number of change points in a time series are typically unknown beforehand, the problem can be regarded as a model selection challenge, aiming to identify the best segmentation among all possible outcomes with the aid of different information criteria. For discussions on using information criteria in general model selection, the readers are referred to the recent review paper by [10]. In addition, the nonparametric methods of detecting multiple change points have been a research focus during these years. Refs. [11,12] focused more on the hypothesis testing method for MCP, while [13,14,15] emphasized more on the idea of model selection. Nevertheless, we will restrict our interests to the parametric set-up and the corresponding log-likelihood and information criteria, which are well-defined.

In summary, the primary objective of this article is to present a selective review of information criteria-based offline MCP detection methods and their practical applications. Our aim is to provide practitioners who require change point detection with a handbook that guides them in making informed decisions regarding the optimal number and locations of change points. By reviewing various methods and discussing their strengths and limitations, we hope to equip readers with the necessary knowledge to select appropriate approaches for their specific change point detection tasks.

The rest of the paper is organized as follows. Section 2 gives a brief formulation of an MCP model with parameters and discusses the change points problems from the perspective of model selection. Section 3 reviews several information criteria for MCP models and their variants. Section 4 enumerates the real-world applications of the mentioned hypothesis-testing based, information-criteria based MCP methods and the hybrid of the two methods. Simulations and a result comparison of the selected methods are presented in Section 5. A case study on the real data set is presented in Section 6 and Section 7 summarizes the review methods and lists out a couple of challenges faced in this research area.

## 2. Problem Formulation

First of all, we construct the general statistical model of parametric change point detection. Given a time series with *N* observations $X=(X_{1},X_{2},\ldots ,X_{N})$, we assume that the distribution of $X_{i}$ is given in the following form:

$$X_{i}∼f\left(x|\theta_{i}\right),$$

where f(·) is the (joint) probability density function (p.d.f.) and the parameter vector

$$\theta_{i}={\left(\beta_{1,i},\ldots ,\beta_{p,i},\lambda_{p+1},\ldots ,\lambda_{p+q}\right)}^{T}\in \Theta \subset R^{p+q}.$$

With the above formulation, $\lambda ={\left(\lambda_{p+1},\ldots ,\lambda_{p+q}\right)}^{T}$ is unchanged. but $\beta_{i}={\left(\beta_{1,i},\ldots ,\beta_{p,i}\right)}^{T}$ could be different at different time points *i*. A joint p.d.f. is needed if the observed time series is multivariate.

Particularly, the case of normal mean MCP model [16] will be used as the illustrative example throughout this paper. This means that the observation $X_{i}$ follows a Gaussian distribution as follows:

$$X_{i}∼N\left(\beta_{i},\sigma^{2}\right)\;\mathrm{with}\;\mathrm{the}\;p.d.f.\;f\left(x|\beta_{i},\sigma^{2}\right)=\frac{1}{\sqrt{2\pi \sigma^{2}}}\exp\left[-\frac{{\left(x-\beta_{i}\right)}^{2}}{2\sigma^{2}}\right],$$

where the mean $\beta_{i}$ could change over time. and the variance $\sigma^{2}$ remains a constant. One shall notice that $\theta_{i}={\left(\beta_{i},\sigma^{2}\right)}^{T}$ and p=q=1 in this case.

### 2.1. Single CP Model Formulation

We now start with the case of single CP problems. Let τ∈{1,2,…,N} be an unknown time point that separates the time series into two segments $\left(X_{1},X_{2},\ldots ,X_{\tau }\right)$ and $\left(X_{\tau +1},X_{\tau +2},\ldots ,X_{N}\right)$. With a single change point, we have the following general form as

$$X_{i}∼\left\{\begin{array}{c} f(x|\mu_{1},\lambda ),\;1\leq i\leq \tau ,\\ f(x|\mu_{2},\lambda ),\;\tau <i\leq N. \end{array}\right.$$

where $\mu_{1}=\beta_{1}=\cdots =\beta_{\tau }\neq \beta_{\tau +1}=\cdots =\beta_{N}=\mu_{2}$.

If $X_{i}$s are normally distributed with constant variance, the general form can be expressed as

$$X_{i}=\beta_{i}+\epsilon_{i}=\left\{\begin{array}{c} \mu_{1}+\epsilon_{i},\;1\leq i\leq \tau ,\\ \mu_{2}+\epsilon_{i},\;\tau <i\leq N, \end{array}\right.\;\epsilon_{i}∼N\left(0,\sigma^{2}\right)\;i.i.d.,$$

which is essentially a piecewise constant mean function plus normally distributed random noise.

For the single CP model, it is natural to formulate the detection of a single change into a hypothesis-testing framework as

$$H_{0}:\mu_{1}=\mu_{2}\;↔\;H_{a}:\mu_{1}\neq \mu_{2}\;\;.$$

Before making inferences on the location of τ, it is crucial to test whether the mean is constant for the whole time series (no change point) or changes at some point τ (single change point, SCP) based on the classical likelihood ratio test or the *F*-test for a normal sequence. This analysis of the SCP model has been extensively studied by many statisticians [6,17,18].

Nowadays, the analysis of time series data often revolves around the detection of multiple change points, aligning more closely with real-world scenarios. The idea of testing the existence of multiple change points has also been explored. However, extending the idea of the single CP hypothesis testing method to MCP problems is subjected to several constraints. Firstly, conducting hypothesis testing on suspected change points multiple times can be cumbersome. This becomes even more pronounced when the number of change points *K* is unknown; we need to conduct hypothesis testing on a combination of NK possible candidate change points, which is formidable for even moderately large *N* and *K*. In addition, hypothesis testing relies on a subjective judgment of the change points under a user-specified significance level α where the result may be heavily influenced by α. Even with these constraints, some researchers [19,20] still derived MCP methods based on hypothesis and permutation testing.

### 2.2. MCP Model Formulation

As mentioned above, it is difficult to apply hypothesis testing multiple times for the detection of the unknown number of change points, but we can employ the concept of model selection when dealing with multiple change points. For MCP, the decision to incorporate a new potential change point into the existing set of *k* detected change points is analogous to choosing between the current model (consisting of various sub-models on k+1 sub-segments) and a new model (with k+2 sub-models encompassing the newly identified segmentation).

Assume that the parameter vector $\theta_{i}$ is a piecewise constant with abrupt changes at *K* different points as $0<\tau_{1}<\tau_{2}<\cdots <\tau_{K}<N$. With *K* change points, we have the following general form as

(1) $$X_{i}∼\left\{\begin{array}{cc} f(x|\mu_{1},\lambda ), & 1\leq i\leq \tau_{1},\\ f(x|\mu_{2},\lambda ), & \tau_{1}<i\leq \tau_{2},\\ \;\;\vdots \\ f(x|\mu_{K+1},\lambda ), & \tau_{K}<i\leq N. \end{array}\right.$$

We still use the piecewise constant means with normal noises discussed in [16] as a representative of the MCP models. Consider a normally distributed time series with *N* observations $X=(X_{1},X_{2},\ldots ,X_{N})$ with $X_{i}∼N\left(\beta_{i},\sigma^{2}\right)$, where the value of mean parameters θ changes at *K* unknown points. We assume that the mean value $\beta_{i}$ is a piecewise constant with abrupt changes at $\tau ={\left(\tau_{1},\tau_{2},\ldots ,\tau_{K}\right)}^{⊺}$. To simplify the formulation, $\tau_{0}=X_{1}$ and $\tau_{K+1}=X_{n}$ are added to τ and the normal mean MCP model can be presented as

(2) $$X_{i}=\beta_{i}+\epsilon_{i}=\mu_{k}+\epsilon_{i},\;\tau_{k}<i\leq \tau_{k+1},\;\mathrm{for}\;0\leq k\leq K+1,\;\epsilon_{i}∼N\left(0,\sigma^{2}\right)\;i.i.d.\;\;\;.$$

According to whether the number of change points *K* is known or unknown, [21] summarized the MCP methods into two categories. He suggested using a quantitative criterion V(τ,X) to measure the goodness-of-fit of change point models, and the model that achieves a minimum of V(τ,X) should give the best segmentation result. The model selection criterion V(τ,X) is assumed to be the sum of the cost of all the segments as

$$V\left(\tau ,X\right)=\sum_{k=1}^{K+1}c\left(X_{\tau_{k-1}:\tau_{k}}\right)$$

where c(·) is a cost function which measures the goodness-of-fit of the model on the sub-segmentation $X_{\tau_{k-1}:\tau_{k}}=\left(X_{\tau_{k-1}+1},\ldots ,X_{\tau_{k}}\right)$.

In the case of a known number of change points *K*, solving the optimization problem

$$\min_{|\tau |=K}V\left(\tau ,X\right)$$

gives the optimal locations of the *K* change points. When the number of change points is unknown, solely optimizing the cost function will inevitably result in a saturated model, i.e., the model with *N* segments, with each observation of the time series serving as an individual sub-model. Such an outcome lacks meaningful interpretation or utility. Thus, a measurement of the model complexity will be added to the sum of cost V(τ,X) as a penalty function to avoid the issue of over-fitting as

(3) $$\min_{\tau }\left[V\left(\tau ,X\right)+P\left(\tau \right)\right].$$

There are various choices of V(τ,X) and P(τ) in the literature (see the reviews of MCP problems by [16,21]). In the article, with the parametric assumption, the minus twice log-likelihood function is chosen to be the cost function c(·) on each sub-segmentation as the goodness-of-fit measure of change point models. Particularly, with the general parametric model, we have the minus twice log-likelihood function as

$$V\left(\tau ,X\right)=\sum_{k=1}^{K+1}c\left(X_{\tau_{k-1}:\tau_{k}}\right)=\sum_{k=1}^{K+1}\left[\sum_{j=\tau_{k-1}+1}^{\tau_{k}}\log f\left(X_{j}|\theta_{k},\lambda \right)\right]=-2\log L.$$

Here, if the normal mean MCP model is chosen, $f\left(X_{j}|\theta_{k},\lambda \right)=f\left(X_{j}|\beta_{k},\sigma^{2}\right)$ becomes the likelihood of a normal distribution. Using the log-likelihood also ensures the consistency in the derivation of information criteria.

An advantage of the optimization formulation in Equation (3) is that, with suitable cost and penalty functions, the penalized cost function can be minimized by dynamic programming exactly and efficiently [22]. Particularly, the Pruned Exact Linear Time (PELT) algorithm proposed by [22] could even achieve a linear time complexity in the sample size under certain conditions. The PELT algorithm and its variants have been proven to work well with various information criteria as long as the penalty function is given [14,23].

## 3. Information Criteria for MCP

As discussed, if the number of change points *K* is unknown, detecting multiple change points could be cast as a model selection problem where the model with the best segmentation result needs to be selected among all candidate models. Under the framework of the information theory, there exists abundant literature on criteria for model selection. Here, we consider three widely applied information criteria: Akaike Information Criteria (AIC) [24], Bayesian Information Criteria (BIC) or Schwarz Information Criteria (SIC) [25] and Minimum Description Length (MDL) [26]; some variants based on these criteria will be reviewed as well. We will extensively discuss the penalty term associated with each information criterion. By examining the penalty term, this article provides insights into how different information criteria balance the model’s complexity and the quality of the fit.

### 3.1. AIC and Its Variant

AIC, first introduced by [24], is a well-known criterion for model selection initially applied to choosing the most suitable statistical models [27]. The AIC is formally defined as follows

(4) AIC=−2logL+2M

where *M* is the total number of free parameters. The penalty term in AIC is twice the number of the model’s parameters. The basic idea behind AIC is a trade-off between the goodness of fit and the simplicity of models. Generally, adding free parameters in the model will lead to a decrease in the minus log-likelihood function, which means a better goodness of fit. AIC advocates the model with excellent goodness of fit but tries to avoid model over-fitting, thus a penalty term that increases along with the number of parameters is added to the minus log-likelihood to balance the number of model parameters.

Ref. [28] studied how to determine the number of change points via AIC. One shall note that the location of the unknown parameter can also be regarded as an unknown parameter and each segment contributes the same number of free parameters *p* to the penalty. In addition, the number of nuisance parameters λ is a constant as *q*, and it is acceptable to neglect this term in AIC. With slight modification to the AIC penalty term in Equation (4), we have the AIC for multiple change point models

AIC=−2logL+2[(K+1)p+K]

The penalty term of AIC in the MCP models is twice the sum of the number of model parameters (K+1)p from all (K+1) segments and the number of change points *K*. The model with the lowest AIC will be selected, and the number of change points is determined by *K*.

Although model selection via AIC is easy to implement without any subjective judgment and can be applied on various statistical model identification problems, some experiment results [29,30] have shown that AIC tends to overestimate the number of the needed parameters, due to the lack of consideration of uncertainty about parameter values and model forms [31].

Therefore, a modified version of AIC is suggested by [32] by taking the irregularity of change-point models into consideration. Ref. [32] recommended raising the penalty term with the form

mAIC=−2logL+2[(K+1)p+3K]

where *K* is the number of change points, and *p* is the number of model parameters of a single segment. Compared with AIC, the modified AIC advocates models with fewer change points as it considers a larger penalty for the number of change points as 6K.

### 3.2. BIC and Its Variants

Dealing with the problem of choosing the appropriate number of model parameters or the dimensionality of the model equivalently, Ref. [25] proposed an alternative approach for this problem based on utilizing the large-sample limits of the Bayes estimator to estimate the maximum likelihood. BIC (or SIC) is defined as follows:

BIC=−2logL+MlogN,

where *M* and *N* are the total number of unknown parameters and the sample size of observed data, respectively. Loosely speaking, BIC penalizes the goodness-of-fit of a model by the product of the number of model parameters and the number of observations Mlog(N).

Ref. [33] introduced BIC to the problem of estimating the number of change points as

BIC=−2logL+[(K+1)p+K]logN

The mathematical proof of the asymptotic property of the criteria has been thoroughly presented in the work of [33].

The same overestimation issue in AIC happens to BIC, where [34] have illustrated that the classical setting of BIC does not theoretically fit segmentation-related problems. In the context of change point detection, simulation conducted by [35] reflected that both AIC and BIC overestimate the number of change points. Therefore, researchers have proposed many variants of BIC suitable for the change point detection problem with better performance.

For the variants of BIC, there exists a substantial body of literature related to this topic. Ref. [36] suggested a modified BIC for single change point detection models based on the analysis of parameter redundancy. Given a single change point model of time series $X_{1},X_{2},\ldots ,X_{N}$ with density function $f(x_{i}|\mu_{1},\lambda )$ for i≤τ and $f(x_{i}|\mu_{2},\lambda )$ for i>τ, the idea is both $\mu_{1}$ and $\mu_{2}$ can be estimated effectively when the change point lies in the middle of 1 and *N*, and either one of two estimates will be less efficient when the change point is close to 1 or *N*. The modified BIC for a single change point is defined as

$$\mathrm{mBICS}\left(k\right)=-2\log L\left(k\right)+\left[2p+{\left(\frac{2k}{N}-1\right)}^{2}\right]\log N,\;k=1,\ldots ,N-1,$$

where $\log L\left(k\right)=\log L\left({\hat{\mu }}_{1},{\hat{\mu }}_{2},\hat{\lambda },k\right)$ is the sum of log-likelihood functions over two segments.

Ref. [37] further generalized the idea of [36] and obtained a modified BIC in the context of multiple change points. Recall the general parametric MCP model in Section 2, the modified BIC in [37] is defined as

$${\mathrm{mBIC}}_{1}=-2\log L+\left[\left(K+1\right)p+C\sum_{k=1}^{K+1}{\left(\frac{\tau_{k}-\tau_{k-1}}{N}-\frac{1}{K+1}\right)}^{2}\right]\log N$$

where C>0 is a constant which is a tuning parameter. Ref. [37] recommended the constant C∈[1,10].

For the normal mean MCP model with the variance known, ref. [34] proposed another modified BIC with a different penalty term by deriving an asymptotic approximation to Bayes factors. With the assumption that change points are located far enough from each other and $r_{k}=\tau_{k}/N\in \left(0,1\right)$ for 1≤k≤K, the modified BIC of [34] is defined as

$${\mathrm{mBIC}}_{2}=-2\log L+3K\log N+\sum_{i=1}^{K+1}\log\left(r_{i}-r_{i-1}\right).$$

Under the assumption of change-point locations, the author argued that even if the term 3Klog(N) will dominate the term $\sum_{i=1}^{K+1}\log\left(r_{i}-r_{i-1}\right)$ in an asymptotical sense, the term $\sum_{i=1}^{K+1}\log\left(r_{i}-r_{i-1}\right)$ can still be significant due to the slow-growing nature of logN. It is worth noting that the modified BIC mentioned above is derived under the specific setting that the variance $\sigma^{2}$ is known. If the variance is unknown, a more complex version is discussed by [34] as well. For the theoretical discussion, see [38].

In addition to the above modifications on BIC, some variants of the BIC incorporate additional scaling parameters. Ref. [35] introduced a user-adjustable tuning parameter ρ to the penalty term in BIC as the shrinkage Bayesian information criterion

$${\mathrm{sBIC}}_{1}=-2\log L+\rho M\log N.$$

However, the adaptive selection of the tuning parameter ρ is rather involved. Ref. [39] recommended ρ>1 when the number of free parameters is not fixed and tends to diverge as N→∞. In the context of CPD, the number of free parameters is contingent upon the number of segments, which further depends on the length of the time series. Ref. [40] set ρ=loglogN in their simulation study and obtained encouraging experiment results. Similarly, ref. [41] considered a strengthened version of the Bayesian information criterion

$${\mathrm{sBIC}}_{2}=-2\log L+M{\left(\log N\right)}^{\alpha }$$

in the proposed wild binary segmentation algorithm. As the name suggests, the requirement of sBIC_2_ is α>1.

### 3.3. Minimum Description Length

Initially, minimum description length (MDL) was a concept in the information theory field and was first considered by [42], which formed the base of the algorithmic notion of entropy. Refs. [26,43] started to apply MDL for the construction and selection of statistical models by selecting the model that gives minimum MDL. One of the significant advantages of MDL is that MDL situationally tailors for parameters with different natures, while other information criteria (e.g., AIC and BIC) place the same penalty on all the parameters [44,45]. Similar to AIC and BIC, we can write the MDL criteria as a penalized likelihood function

$$\mathrm{MDL}=-2\log_{2}L+P$$

where *L* is the likelihood function, and *P* is the penalty term.

Here, we construct the penalty term in MDL with three principles stated by [46]:

1. The penalty of a real-valued parameter estimated by *n* data points is $\log_{2}n$;
2. The penalty of an unbounded integer parameter *K* is $2\log_{2}K$;
3. The penalty of an integer bounded by a known integer *N* is $2\log_{2}N$.

Recall the formulation of the general parametric model in Section 2, the MDL penalty terms of all the unknown parameters are given below:

$$\begin{array}{cc} \; & P_{\mu }=p\sum_{i=1}^{K+1}\log_{2}\left(\tau_{i}-\tau_{i-1}\right)\\ \; & P_{\lambda }=q\log_{2}N\\ \; & P_{K}=2\log_{2}K\\ \; & P_{\tau }=2K\log_{2}N \end{array}$$

Collecting all above MDL penalty terms and eliminate constant terms irrespective of changes in the number and positions of change points, the MDL criteria for MCP models is

$$\mathrm{MDL}=-2\log L+2\log K+2K\log N+p\sum_{i=1}^{K+1}\log\left(\tau_{i}-\tau_{i-1}\right)$$

Please note that a slight revision of the base logarithms has been made to the penalty term in the aforementioned MDL formula. Replacing the base two logarithms with natural logarithms does not impact the condition where the minimum is attained.

## 4. Review on Applications

### 4.1. Application of Hypothesis-Testing Based Methods

In this section, our objective is to revisit the practical applications of hypothesis-testing methods for detecting change points. As we referred to in Section 2, conducting hypothesis tests at suspected points is a standard approach in single CP problems. However, when confronted with large quantities of candidate sets of multiple change points, several limitations arise, restricting the repeated use of hypothesis testing for exhaustive comparison. Consequently, the application of hypothesis testing-based CP methods is predominantly confined to scenarios where the time series contains only one or two change points.

Ref. [47] designed a robust weighted partial *F*-test procedure to solve the situation of one and two change points in the piecewise linear regression model with Gaussian innovation, and applied their methods to the analysis of change of stagnant band height data and three attributes of a plant’s organ. Both situations where the location of the change point is known or unknown are discussed. A straightforward comparison between the *F* statistics and the critical value solves the former case, while a grid search with hypothesis testing is proposed as the solution of the latter case. The concept of utilizing the *F*-test was also implemented in the tire industry by [48]. The footprint pressure curve of tires is a bathtub-shaped curve with two change points. The author applied the *F*-test on the model parameter for the selection of models of two change points, which is to choose one from a model with three straight lines and a model with two straight lines and a quadratic curve in the middle.

For tackling MCP problems, ingenious designs incorporating hypothesis testing and other methodologies have been employed by researchers to identify and detect multiple change points. In an interesting research study focused on analyzing change points in terrorism-related online content [49], the author employs a combination of hypothesis testing, the permutation test, and the concept of binary segmentation. Raw inputs gathered are categorized by the CNN-based classification model, and the empirical divergence measurement of the sample is calculated as the detection criterion of a single change point. The procedure involves verifying whether a suspected point represents a genuine change point by comparing the test statistics obtained after a substantial number of permutations with the approximate *p*-value. If the estimated change point is deemed statistically significant, the time series is divided into two parts, and the procedure is repeated until no further significant change points can be identified. Similarly, ref. [50] adopted hypothesis testing combined with parametric bootstrapping in their proposed method for recurrent-event change point analysis, aiming to determine the number of clusters of change points in the UK coal mining disaster data.

### 4.2. Application of Information Criteria Based Methods

In this section, we aim to look back at the applications of information criteria based on MCP methods. There is a rich literature in academia on utilizing information criteria for model selection in the MCP field, especially for those methods that adopt and extend the idea of binary segmentation. Some research considered reforming the MCP from finding the global extremum value of the cost function to an optimization problem in some sub-interval, as computation cost is reduced dramatically when solving MCP locally. Generally, such methods will overestimate the number of change points since introducing more change points always leads to a reduction in the sum of cost. Therefore, information criteria are often introduced as the penalty of model complexity (more change points make a more complex model) to avoid overfitting. In the study of the changes in the UK house price index and COVID-19 outbreak time series, ref. [51] adopted the sBIC_2_ to determine the optimal number of change points in the mean shift model with a normally distributed error.

In the area of biostatistics, most researchers are drawn to the MCP in the mean shift model with Gaussian innovation. The Screen and Ranking algorithm (SaRa) suggested by [52], which is an MCP approach via local information, applies BIC and mBIC_2_ to decide the suitable number of changes in the DNA copy number variation data stream. There are many other studies following the same mean shift model setting using BIC to determine the change points number, such as the examination of array comparative genomic hybridization utilizing sBIC_1_ [40] and the analysis of gene copy number using BIC [53]. In contrast to the mean shift model, ref. [54] explored change points in the data of psychopathology patients, specifically an auto-regressive time series of order 1, aka AR(1), with Gaussian innovations. The study employed AIC and BIC to determine the number of regimes.

Meteorology is another field that extensively uses information criteria for MCP analysis. In the study of the precipitation data in Hebei province (China) [55] and the nitrogen oxide concentrations data in London [56], BIC is adopted for selecting the optimal number of change points. The study by [55] assumed that the observation of precipitation data follows a binomial distribution, while ref. [56] simply employed the mean shift model but did not explicitly specify the distribution of the white noise. In addition to BIC, MDL is also widely used in this field; ref. [44] applied MDL to gauge the number of change points and their locations in a lognormally distributed temperature series from 1901 to 2000 in Tuscaloosa. Moreover, ref. [57] utilized MDL to study change points in multivariate normally distributed data and validated the effect using precipitation data from 1818 to 1990 in New Bedford and the North Atlantic tropical cyclone record. For the comparison between AIC, BIC, and MDL in this periodic auto-regressive time series, ref. [58] delved into the MCP problem for the flow data of two real rivers (the South Saskatchewan River and the Colorado River). The analysis takes into account various factors such as seasonality and changes in reservoirs or other hydrological facilities. The study explored the effectiveness of AIC, BIC, and MDL during the process of model training, and the result shows that BIC and MDL always detect the correct number of change points, while AIC sometimes has the issue of over-estimation.

Additionally, several application examples of information criteria-based MCP model selection in the field of engineering are presented below. In the manufacturing domain, the detection of change points plays a crucial role in phase I analysis, also known as retrospective analysis in statistical process control. The investigations conducted by [59,60] focused on clustering manufacture-process time series of multivariate normal distribution and regular/mixed polynomial models into distinct independent components. The analysis of time series is subsequently transformed into the analysis of these components, with the recommended number of components determined by AIC and MDL. For determining the number of change points in the Body in White (a stage in automobile manufacturing) time series, ref. [61] has considered conducting a cumulative difference contribution selection with a threshold of 0.8 (according to the Pareto principle) for the initial change points selection, followed by a BIC based model selection for change points trimming. Without the loss of generality, the author formulated the time series in a piecewise linear regression model and tested four types of noise with zero means (Gaussian noise, *t*-distributed noise, lognormal noise, and the mixed noise of t and lognormal). Furthermore, in the analysis of structural breaks and change points in panel data time series, ref. [62] tested the effect of AIC and BIC in determining the number of breaks. Interestingly, the results given by [62] showed the performance of AIC surpasses BIC, probably due to a lack of sufficient observations in the tested time series.

### 4.3. Appliction of Hybrid Methods

In this section, we will review the applications that adopt a hybrid of hypothesis testing and information criteria. A pioneering work exploring hybrid MCP methods on real-world data is found in the research on change points for stock prices [63]. This study employed hypothesis testing to examine the presence of change points under a Gaussian prior distribution. The null hypothesis $H_{0}$ assumed a model without any change points in the variance of stock prices, while the alternative hypothesis $H_{a}$ proposed the existence of *K* change points. Diverging from classical hypothesis testing, the test statistics utilized the minimum of the BIC for all possible multiple change-point models, denoted as

$$\mathrm{BIC}\left(\hat{k}\right)=\min_{1\leq k<K}\mathrm{BIC}\left(K\right)$$

The critical value, on the other hand, was determined by the BIC of the model with no change point BIC(0). If $\mathrm{BIC}\left(0\right)\leq \mathrm{BIC}\left(\hat{k}\right)$, we reject $H_{0}$. When the BIC under $H_{0}$ is very close to the BIC under $H_{a}$, to verify whether it is caused by the fluctuation in the data or the influence of the change point, the author introduced the significance level α with the associated critical value $C_{\alpha }>0$ and reject $H_{0}$ if $\mathrm{BIC}\left(0\right)\leq \mathrm{BIC}\left(\hat{k}\right)+C_{\alpha }$. The concept introduced by [63] has seen widespread application across diverse disciplines, with various prior assumptions about data distribution. This includes the use of BIC in studying normally distributed water quality data [64] and analyzing a fleet of wind turbines with Poisson-distributed failures [65], the use of mBICS for MCP on Weibull-distributed rainfall data [66], and the application of mBIC_1_ in the calibration of a force balance used in NASA’s wind tunnel experiment [67] as well as the examination of stock market data under skew-normal distribution [68]. It is worth noting that [65] simply borrowed the idea of using BIC as a statistical test but did not consider the significance level, test statistics, and critical value in detail.

Another type of hybrid MCP method is explored in the study of wind speed simulation using historical data [69]. The methodology involves conducting repeated significance tests on suspected change points to identify genuine change points that effectively partition the historical wind speed data into sub-segments. Subsequently, an Auto-Regressive (AR) model with an unknown order, denoted as *p*, is constructed and fitted to each sub-segment. In [69]’s work, the AIC is employed to determine the optimal order of the AR model. Analogous utilization of AIC can be found in the research of shake table tests (for seismic provision) carried out by [70].

The literature review on the applications of MCP is summarized in Table 1.

## 5. Simulation Study

In this section, we conduct simulation studies to investigate the performance of various information criteria in detecting multiple change points. To ensure the estimability of model parameters, we assume a minimum of two observations for each segment. All simulation studies presented in this section are grounded in the normal mean MCP model outlined in Section 2, with a unit constant variance $\sigma^{2}=1$ for simplicity. For readers interested in the scenario involving changing variance under constant mean, as well as changes in both mean and variance, a brief exploration with discussion is provided in the Appendix A and Appendix B. We simulate the time series using three different distributions, i.e., (1) normal distribution, (2) log-gamma distribution, (3) auto-regressive process of order 1, aka AR(1) with Gaussian innovation. Different data-generating processes are utilized to assess the efficacy of using information criteria based on normal distribution in detecting change points with possible model mis-specification. The selection of auto-regressive coefficients is ϕ=±0.5. We investigate the detection power and accuracy of different information criteria, including AIC, mAIC, BIC, mBIC_1_, mBIC_2_, and MDL. Since mBICS is proposed for detecting a single change point, we ignore mBICS in the simulation study. Additionally, we exclude sBIC_1_ and sBIC_2_ due to the necessity of selecting the tuning parameter. Interested readers can refer to the works of [39,41] for guidance on optimal tuning parameter selection. The notation $\left(\mu_{1},\sigma^{2}\right)\overset{\tau }{⟶}\left(\mu_{2},\sigma^{2}\right)$ is used to denote the transfer between the vector of model parameters after the τth observation [23]. All of the simulations are run by Python 3.11 with the package *ruptures*, and the PELT algorithm [22] is applied to search the change points. Throughout the simulation in this section, no specific constraint is imposed on the minimum distance between change points during the execution of the PELT algorithm. Additionally, the required parameter *min_size* for the built-in PELT algorithm is set to be 2 by default.

### 5.1. Simulation on Different Magnitude of Mean Shifts

We first explore the performance of selected information criteria under different mean shift magnitudes. The number of change points *K* is fixed to be 8, and the length of 9 segments is generated by

$$\left(L_{1},L_{2},\ldots ,L_{9}\right)∼50+\mathrm{Multinomial}\left(50\cdot 9,p_{1},\ldots ,p_{9}\right),$$

where $\left(p_{1},p_{2},\ldots ,p_{9}\right)$ adheres to a uniform Dirichlet distribution with a constant concentration parameter α=1. The generation of the segment length assures a lower bound of 50 for each segment while making them randomly different lengths. The locations of change points are given by

$$\tau_{j}=50j+\sum_{i=1}^{j}L_{j},j=1,\ldots ,8.$$

The mean shift pattern for the normally distributed time series is set as follows

$$\left(\mu ,\sigma^{2}\right)\overset{\tau_{1}}{⟶}\left(\mu +\Delta \mu ,\sigma^{2}\right)\overset{\tau_{2}}{⟶}\left(\mu ,\sigma^{2}\right)\overset{\tau_{3}}{⟶}\cdots \overset{\tau_{7}}{⟶}\left(\mu +\Delta \mu ,\sigma^{2}\right)\overset{\tau_{8}}{⟶}\left(\mu ,\sigma^{2}\right).$$

We perform the simulation with the initial mean μ equal to 1 and mean shift magnitudes Δμ ranging from 0.25 to 2, with an incremental step of 0.25 in each run. In the scenario involving the log-gamma distribution and AR(1) with Gaussian innovation, a suitable parameter transformation is employed to ensure that all the time series exhibit identical mean shift magnitude and a constant variance of 1, as set in the normally distributed time series.

**Figure 1:**
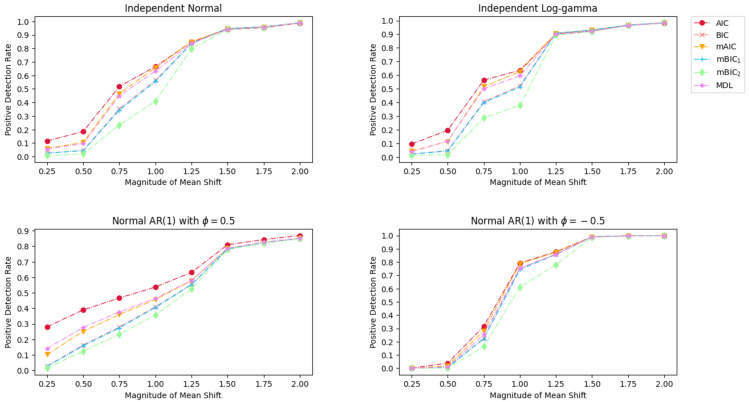
Simulation results of Positive Detection Rate for various magnitude of mean change under different distributions.

In the simulation study, a margin of 5 is selected as the tolerance of detection fault. If a detected change point is located in the interval [τ−5,τ+5], we regard this point as a correctly detected change point. The positive detection rate (PDR) defined as follows

$$\mathrm{PDR}=\frac{\hat{k}}{k},$$

is calculated to evaluate the effect of mean shift magnitude on the performance of selected information criteria, where $\hat{k}$ is the number of the correctly detected change points, and *k* is the number of the true change points. The PDR for the three distribution set-ups using a number of Monte Carlo 1000 replications is illustrated in Figure 1.

For the upper two cases of Figure 1 where the time series follows normal and log-gamma distributions, a shift magnitude of Δμ=1.25 yields favorable detection results, with PDR for all information criteria nearly surpassing 0.8. However, the results of the AR(1) time series show a contratic effect. When AR(1) coefficient ϕ=0.5, a mean shift of 1.75 is necessary to guarantee that the PDR exceeds 0.8 for all methods. Conversely, only a mean change of 1.0 is needed when ϕ=−0.5. It can be observed that BIC and mBIC_1_ yield very similar PDR values, as evidenced by the overlapping curves. Similarly, the outcomes of MDL and mAIC are closely aligned since their curves highly coincide with each other. In addition, one can see that AIC, mAIC, and MDL achieve a higher PDR than BIC and its variants. This is attributed to the fact that the BIC family imposes more stringent penalties on model complexity compared to the AIC family. Consequently, a larger mean shift magnitude is necessary for BIC and its variants to substantiate the presence of a suspicious change point.

**Figure 2:**
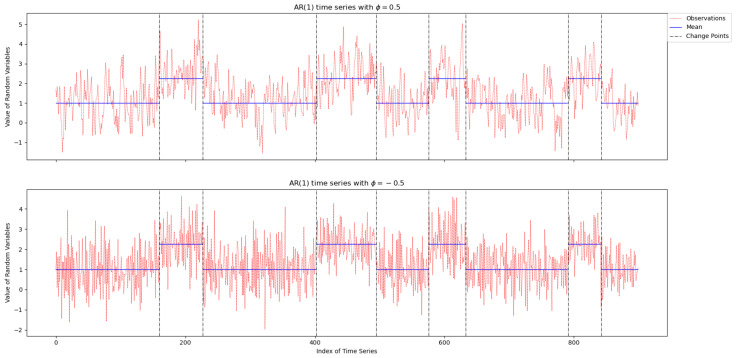
Simulated AR(1) time series with mean (blue solid line) and change points (black dot line) where k=8 and Δμ=1.25.

Figure 2 shows a simulation path of the time series under AR(1) with Gaussian innovation. The results show that when the auto-regressive coefficient ϕ is positive, there exist local sharp increase and decrease processes in the time series, which makes the detection of change points more difficult. If the local increase or decrease trends occur around the location of the mean shift, the true change point will probably be masked off. On the contrary, the value of the time series fluctuates evenly near the mean value and makes the detection of change points easier when ϕ is negative.

### 5.2. Simulation on Different Number of Change Points

The findings from the previous section imply that information criteria related to BIC require a broader range of mean changes to achieve effective detection results. In this subsection, the magnitude of the mean shift is kept constant, but the number of change points increases with the length of the time series. We opt for an initial mean μ equal to 1 and a fixed mean shift of 1.25, as suggested by the results of PDR curves in the previous section. Similar to the segment length generation used before, let the number of change points *K* rise from 1 to 20, and the length of the K+1 segments is generated by

$$\left(L_{1},L_{2},\ldots ,L_{k+1}\right)∼50+\mathrm{Multinomial}\left(50\cdot \left(k+1\right),p_{1},\ldots ,p_{k+1}\right).$$

Three criteria are adopted to measure the performance, namely, the precision rate, the recall rate, and the ratio of change point numbers. Let $\hat{k}$, *k*, and $\tilde{k}$ be the number of detected change points, the number of true change points, and the number of correctly detected change points. The precision score is defined as

$$\mathrm{Precision}\;\mathrm{Rate}=\frac{\tilde{k}}{\hat{k}}.$$

The recall score is defined as

$$\mathrm{Recall}\;\mathrm{Rate}=\frac{\tilde{k}}{k},$$

and the ratio of change point numbers is calculated by

$$\mathrm{Ratio}=\frac{\hat{k}}{k}.$$

**Figure 3:**
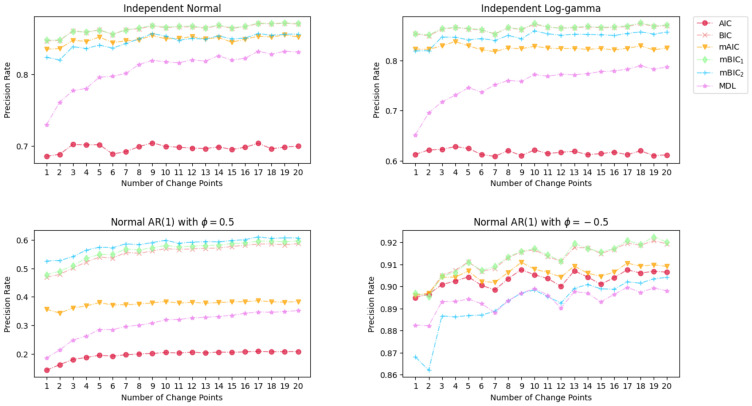
Simulation results of Precision Rate for various magnitude of mean change under different distributions.

**Figure 4:**
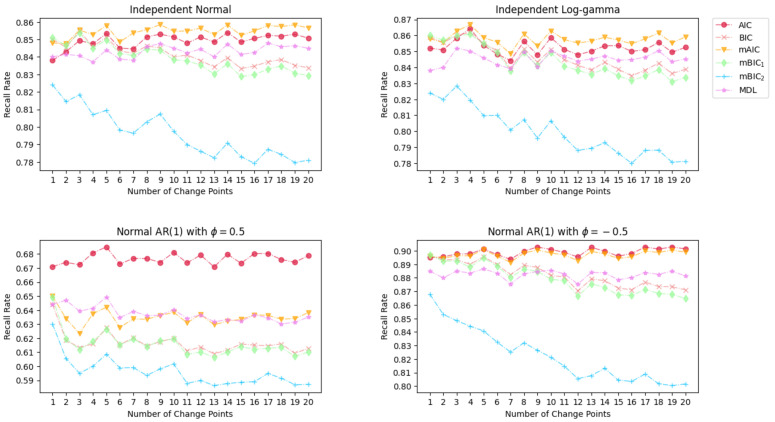
Simulation results of Recall Rate for various magnitude of mean change under different distributions.

**Figure 5:**
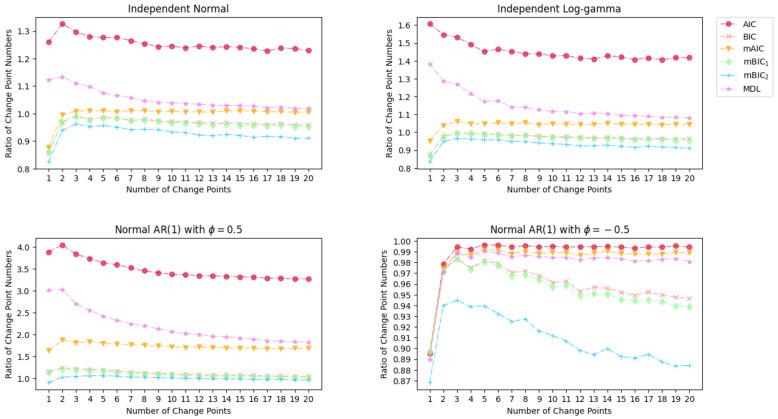
Simulation results of Ratio of Change Point Numbers for various magnitude of mean change under different distributions.

The results of the Monte Carlo simulation with 1000 times replication on the precision rate, recall rate, and the ratio of change point numbers are shown in Figure 3, Figure 4 and Figure 5.

Figure 3 illustrates that the precision rates provided by BIC, mBIC_1_, mBIC_2_, and mAIC are closely aligned and surpass the precision offered by AIC and MDL when the times series follows the distribution of normal, log-gamma, or normal AR(1) with a positive coefficient. The upper two panel shows over 80% of the identified points from the BIC family and mAIC are accurate. There is a slight improvement in the precision rate of MDL when the number of change points rise from 1 to 10 and finally converges to 0.8 and 0.78, respectively. AIC exhibits the least favorable performance among the mentioned criteria, with precision rates at approximately 0.7 and 0.6. For the bottom two cases, when ϕ=0.5, the performance of all information criteria is notably poorer compared to their performance in the aforementioned two cases. However, their precisions are closely clustered in the range of 0.86 to 0.92 when ϕ=−0.5. It is noticeable that mBIC_2_ gives very high precision rates except for detecting change points in time series with a negative AR coefficient.

The results depicted in Figure 4 reveal that mBIC_2_ consistently yields the lowest recall rate across all cases. This can be attributed to mBIC_2_’s imposition of higher penalties of 3logN on newly emerging change points, leading to the detection of only those change points that significantly reduce the Maximum Likelihood Estimate (MLE). For a fixed mean shift magnitude and constant variance, the penalty given by mBIC_2_ seems a little bit excessive. For the normal and log-gamma distribution scenarios, all the information criteria despite mBIC_2_ show a relatively acceptable recall rate over 0.83, and the performance of AIC with its modified version mAIC slightly overtakes other information criteria. The outcome for the time series with AR(1) and Gaussian innovation aligns with the results presented in Figure 2. When ϕ=0.5, the presence of local upward and downward trends complicates the detection of change points, resulting in a deterioration in the recall rates for all the examined information criteria compared to the cases with normal and log-gamma distributions. Conversely, for a negative ϕ=−0.5, the situation is reversed, and the high recall rates indicate that the submodels selected by these tested information criteria suitably capture the characteristics of the time series.

It appears that AIC, mAIC, and MDL offer superior change point models, as evidenced by their higher recall rates compared to BIC, mBIC_1_, and mBIC_2_. However, the ratios of change point numbers, as represented in Figure 5, indicate that AIC and MDL tend to overestimate the number of change points when the underlying distribution of the time series follows a normal or log-gamma distribution. In the case of normally distributed time series, mAIC and MDL accurately estimate the correct number of change points, whereas BIC, mBIC_1_, and mBIC_2_ slightly underestimate the number of change points, and AIC tend to suffer from overestimation. Upon a shift to a log-gamma distribution in the underlying data, the BIC family provides a more precise estimation of the change point number, while the models selected by AIC, mAIC, and MDL tend to overestimate the number of change points. Similarly, when dealing with time series featuring a positive AR coefficient, the performance of AIC, MDL, and mAIC degrades, while the BIC family continues to provide accurate estimations of the number of change points. Additionally, when the time series has a negative AR coefficient, all criteria outperform their performance in the other three cases.

## 6. Case Study

In this section, time series from real datasets are investigated to compare the detection effect of the selected information criteria. Specifically, we focus on identifying change points in the SCADA signals of wind turbines. Detecting these change points during the operation of wind turbines is crucial for preemptively addressing potential incidents before they escalate into more serious events. In addition, it offers valuable insights for routine inspection and maintenance planning. The SCADA data of wind turbines is taken from [71]. It consists of 11 proposed SCADA signals of a wind turbine. The sample rate of the original data is in a typical 10 min resolution, and then the signals are averaged each day after proposing. Since this dataset has been discussed by [71] in detail, we only present a brief investigation of it.

**Figure 6:**
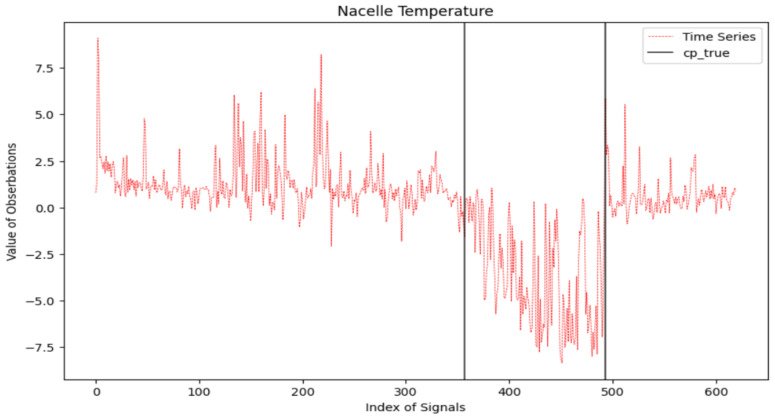
Nacelle temperature signals with two labeled change points (in vertical solid black lines).

**Figure 7:**
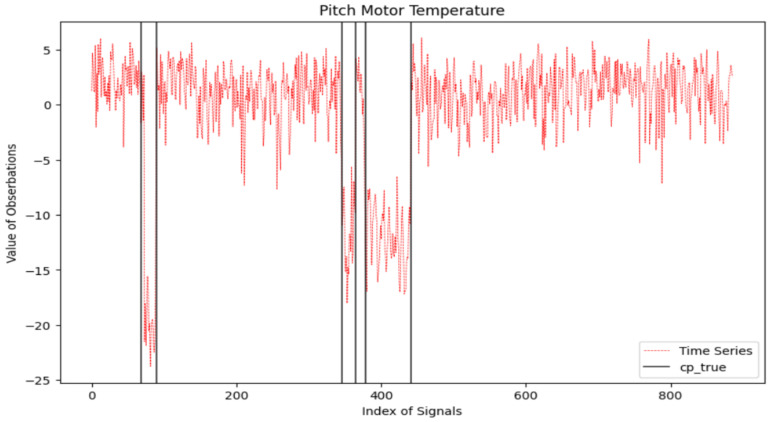
Pitch motor temperature signals with six labeled change points (in vertical solid black lines).

We analyze and apply the information criteria penalized MLE to the signals of nacelle temperature (collected from 1 January 2017 to 12 September 2018, spanning a total of 620 days) and the pitch motor temperature (collected from 1 January 2017 to 6 June 2019, spanning a total of 886 days). Given the limited prior knowledge we have about these two signals, we make the assumption that the time series follows a normal distribution, and both mean and variance changes may occur in the two signals. Figure 6 and Figure 7 display the two signals. The 357th and 493rd observations in the nacelle temperature signals and the 68th, 90th, 346th, 364th, 379th, and 441st observations in the pitch motor temperature are labeled as the change point.

**Figure 8:**
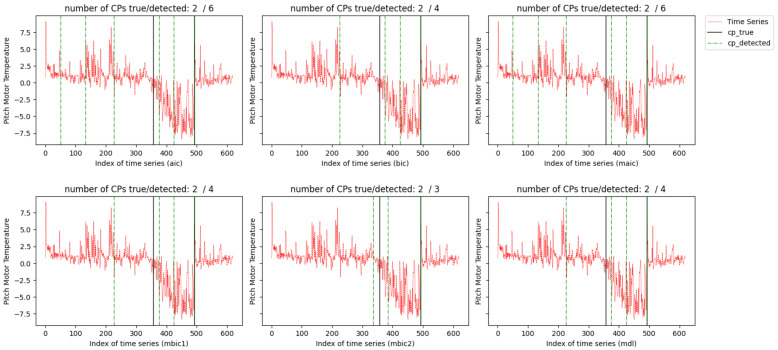
Nacelle temperature signals with two labeled change points (in vertical solid black lines).

**Figure 9:**
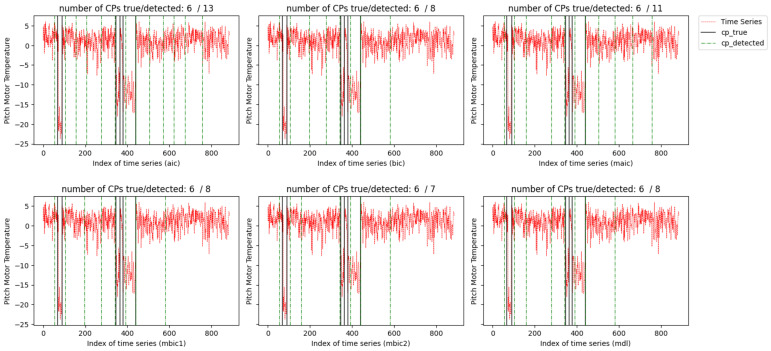
Pitch motor temperature signals with six labeled change points (in vertical solid black lines).

The results of tested methods under the setup of d=50 are plotted in Figure 8 and Figure 9. Considering the nacelle temperature signal, all six information criteria accurately identify the 493th observation as a change point. For the detection of the change point occurring at the 357th observation, mBIC_2_ gives the closest detection outcome. BIC, mBIC_1_, and MDL incorrectly classify the 226th and 425th observations as change points, while AIC and mAIC exhibit more severe false alarms. For the pitch motor temperature, some change points are located very close, and the variance in the signal is larger than in nacelle temperature. Thus, the detection effects of all methods deteriorate. Similarly, mBIC_2_ achieves the most accurate detection, followed by BIC, mBIC_1_, and MDL. AIC shows the worst outcome with many false alarms.

## 7. Discussion, Summary and Future Perspective

In this article, we consider the detection of an unknown number of change points as a model selection problem. We provide a comprehensive review of various information criteria and conduct simulations to evaluate the effect of different information criteria for the selection of submodels. We start formulating the MCP problem from the detection of a single change point, where hypothesis testing is heavily used in this scenario. Then we present the MCP problem and conduct simulations to test the reviewed information criteria following the idea of a normal mean multiple change point model in [16], where the time series is assumed to exhibit sudden variations in mean while maintaining a constant variance. We also conduct the case study on MCP in SCADA signals of wind turbines to provide a concise illustration of the potential to use these information criteria for real-world problems.

We mainly reviewed three types of information criteria: (1) AIC and modified AIC, (2) BIC and modified BIC, and (3) MDL. The applications of these criteria are reviewed in Section 4. Generally, information criteria are often added to penalize the cost function to avoid over-estimation of change points, while some researchers combine hypothesis testing and information criteria to develop a hybrid MCP approach. To be mentioned, some other model selection criteria such as Takeuchi Information Criteria (TIC) [72], Network Information Criteria (NIC) [73], Deviance Information Criteria (DIC) [74] and Integrated Completed Likelihood (ICL) [75] attract less attention in MCP but could be possible choices for future research.

Based on the assumption of the normal mean MCP model, the results of the simulation study in Section 5 illustrate that when the prior assumption that the time series follows normal distribution is wrong, using the MLE of the normal distribution as the cost function with the penalization of information criteria still works, except for the case when there exists a dependence structure in time series when the auto-regressive coefficient is positive. In summary, AIC and MDL often overestimate the number of change points, while mBIC_2_ suffers from underestimation. The model selected by mAIC, BIC, and mBIC_1_ gives the precise estimation of the change point number when there is no auto-regressive relationship in the time series. Since there is no big difference between the overall performance of BIC and mBIC_1_, we suggest that users with practice purposes use mAIC and BIC for simplicity, and use only BIC when there exists strong evidence of auto-regressive relation in the time series.

When using these information criteria in practical applications, the efficacy of these information criteria is significantly influenced by the inherent properties of real data, such as seasonality, trend, or strong noise level. Having prior knowledge in the field or the assistance of experts could greatly improve the detection of MCP.

Although MCP with information criteria was proposed many years ago and significant progress has been achieved in the last two decades, many open challenges still persist in the field.

The first challenge is to deal with non-stationary time series where the distribution or dependence structure of the time series may change right after certain points. As information criteria penalties are typically contingent on both the number of change points and model parameters, any variation in the distribution model can compromise the performance of information criteria, leading to issues of overestimation or underestimation.

Another issue is the high-dimensional time series. The principle of using information criteria for MCP is maximum likelihood estimation under model complexity constraints. However, in the context of high-dimensional time series, calculating the required statistical quantities such as the variance/covariance matrix of the time series can be computationally costly. Finding some robust replacement or approximation to those conventional statistical quantities may provide a good solution.

Lastly, numerous MCP methods identify change points within pre-defined sub-segments, guided by the principle of locality. Using data in proximity to the change point often yields more accurate results than analyzing the entire time series, as observations distant from the change point can introduce bias in the detection outcome. Nevertheless, in instances where the magnitude of change is not strong enough, detecting such changes within a local sub-segment proves challenging due to an inadequate number of samples for inference. An algorithm that dynamically adjusts the length of sub-segment generation based on signal strength may yield superior results, even there are overlaps between sub-segments occur.

## Figures and Tables

**Table 1:** Summary of reviewed applications.

Publication	Number of CPs	MCP Formulation	Model Selection Criteria
[47]	one or two	Piecewise linear regression with Gaussian noise	hypothesis testing
[48]	two	Piecewise linear or quadratic regression	hypothesis testing
[49]	multiple	Empirical divergence measure	hypothesis testing
[50]	multiple	Recurrent *K*-means cluster model	hypothesis testing
[51]	multiple	Mean shift model with Gaussian noise	sBIC_2_
[52]	multiple	Mean shift model with Gaussian noise	BIC, mBIC_2_
[53]	multiple	Mean shift model with Gaussian noise	BIC
[40]	multiple	Mean shift model with Gaussian noise	sBIC_1_
[54]	multiple	Mean shift model with Gaussian noise	AIC, BIC
[55]	multiple	AR(1) with multivariate Gaussian innovation	BIC
[56]	multiple	Mean shift model with noise	BIC
[44]	multiple	Periodic linear regression with noise	MDL
[57]	multiple	Lognormal distribution	MDL
[58]	multiple	Periodic AutoRegressive model	AIC, BIC, MDL
[59]	multiple	Multivariate normal distribution	MDL
[60]	multiple	Regular/Mixed-effect polynomial model	AIC, MDL
[61]	multiple	Piecewise linear regression with noise	BIC
[62]	multiple	Mean shift model with noise	AIC, BIC
[63]	multiple	Normal distribution	Hybrid (BIC)
[64]	multiple	Normal distribution	Hybrid (BIC)
[65]	multiple	Poisson distribution	Hybrid (BIC)
[66]	multiple	Weibull distribution	Hybrid (mBICS)
[67]	multiple	Piecewise linear regression with Gaussian noise	Hybrid (mBIC_2_)
[68]	multiple	Skew normal distribution	Hybrid (mBIC_2_)
[69]	multiple	Vector auto-regressive model with Gaussian innovation	Hybrid (AIC)
[70]	multiple	Single-variate auto-regressive model	Hybrid (AIC)

**Figure A1:**
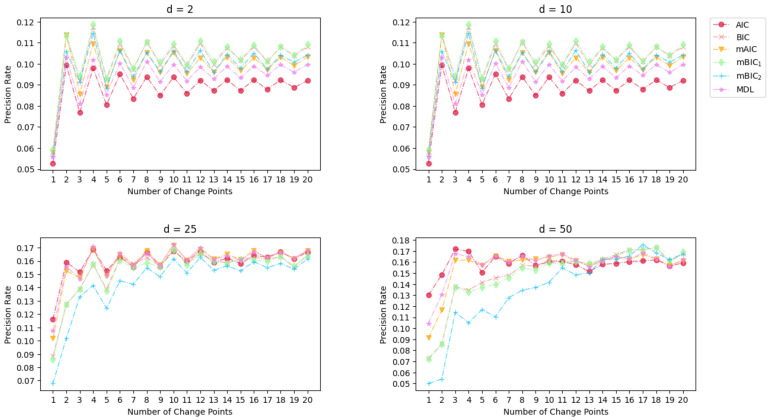
Simulation results of Precision Rate for variance shift time series under different minimum segment distance.

**Figure A2:**
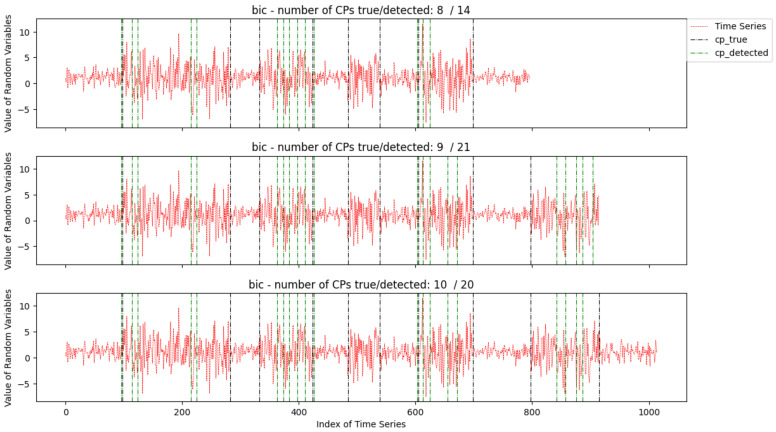
Example for the discussion of fluctuation in precision rates in variance shift time series.

**Figure A3:**
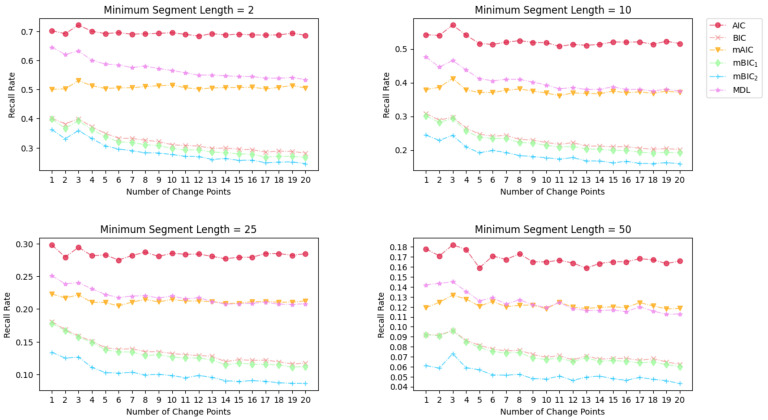
Simulation results of Recall Rate for variance shift time series under different minimum segment distance.

**Figure A4:**
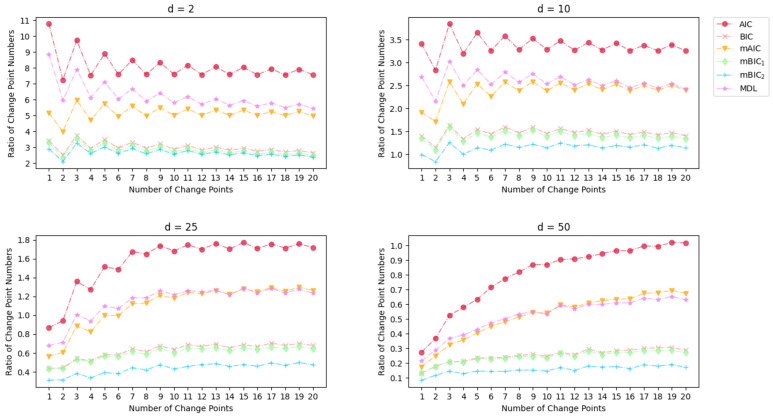
Simulation results of Ratio of Change Point Numbers for variance shift time series under different minimum segment distance.

**Figure A5:**
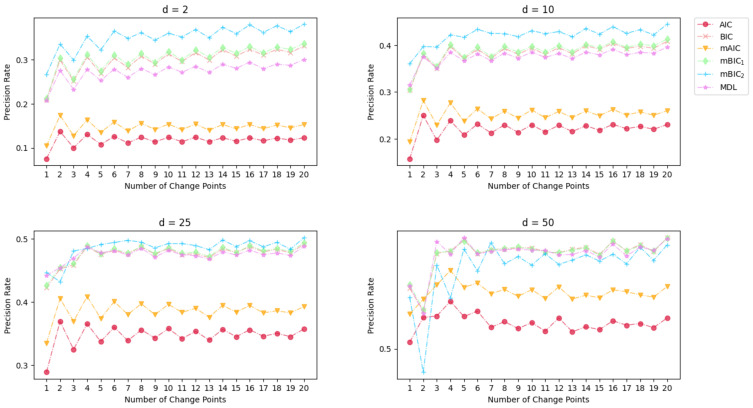
Simulation results of Recall Rate for variance shift time series under different minimum segment distance.

**Figure A6:**
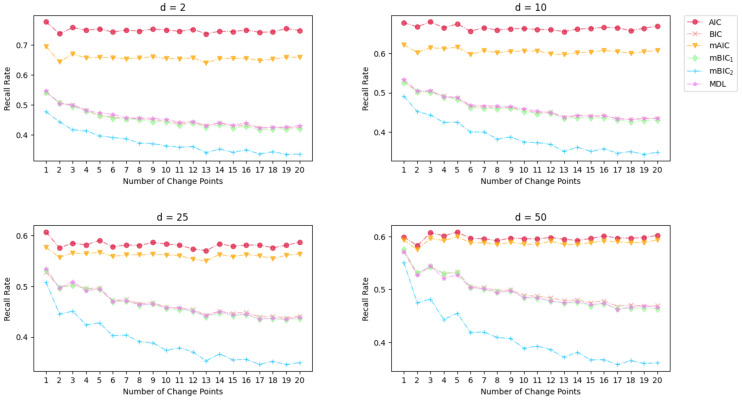
Simulation results of Ratio of Change Point Numbers for variance shift time series under different minimum segment distance.

**Figure A7:**
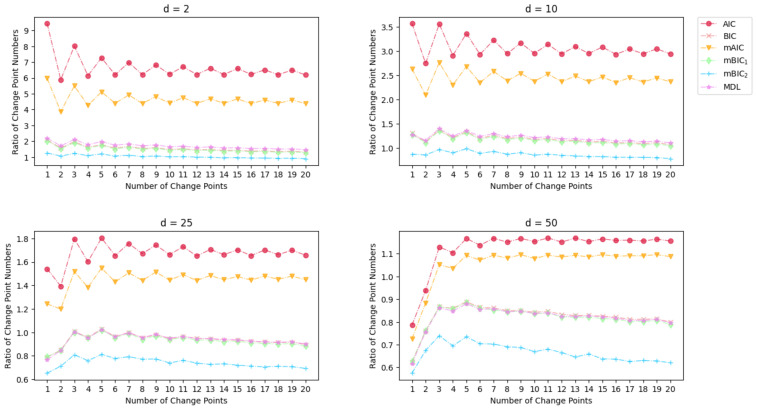
Simulation results of Ratio of Change Point Numbers for variance shift time series under different minimum segment distance.

## Data Availability

No new data were created or analyzed in this study. Data sharing is not applicable to this article.

## Appendix A. Simulation and Discussion for Variance Change Case

In this section, we will present and explain the simulation result of the case where variations solely occur in the variance of the time series observations while the mean μ remains 1. For simplicity, we test the normally distributed time series only.

Following the same time series generation principle in Section 5, let the number of change points rise from 1 to 20 with the variance shifting pattern

$$\left(\mu ,\sigma^{2}\right)\overset{\tau_{1}}{⟶}\left(\mu ,\sigma^{2}+\Delta \sigma^{2}\right)\overset{\tau_{2}}{⟶}\left(\mu ,\sigma^{2}\right)\overset{\tau_{3}}{⟶}\ldots \overset{\tau_{19}}{⟶}\left(\mu ,\sigma^{2}+\Delta \sigma^{2}\right)\overset{\tau_{20}}{⟶}\left(\mu ,\sigma^{2}\right).$$

where the initial variance is $\sigma^{2}=1$, and the variance change magnitude $\Delta \sigma^{2}$ is 8 ($\Delta \sigma^{2}=8$ is a significant change in this case based on testing). Different from the simulation of normal mean MCP where no configuration for the minimum distance *d* between change points is necessary, in the variance change scenario, the minimum distance *d* between change points becomes a crucial parameter when employing MLE with information criteria for change point detection. In instances where the variance experiences a sharp increase, insufficiently large values of *d* can lead to the generation of numerous closely spaced change points due to the substantial dissimilarity in the data. This occurs because the short sub-segments, delineated by these change points, contribute to a decrease in the loss function that far surpasses the increase in the penalty term of the information criterion. We test the defection effect of selected information criteria with four different d=2,10,25,or50.

The results of the Monte Carlo simulation with 1000 times replication on the precision rate for variance change case is shown in Figure A1.

When the minimum distance between change points is small (d=2,10), the poor precision rates given by all of the tested information criteria indicate that there are too many misidentified change points. However, when *d* increases to 25 and 50, there is only a minor improvement in the precision rates. This is because only a few change points that lead to a significant decrease in the cost function will be found when *d* is large, causing low precision rates. It should be noticed that when d=2,10,or25, all precision rates keep fluctuating up and down. We illustrate this phenomenon using a sample time series path (shown in Figure A2) with the number of change points changing from 8 to 10 and d=10.

As the number of change points increases from 8 to 9, the additional segment of data introduces a significant variance, resulting in the detection of numerous closely spaced false change points. Consequently, when the number of change points increases from 9 to 10, the newly added segment comprises data with a small variance, thereby reducing the overall dissimilarity of the time series. This reduction in dissimilarity contributes to a decrease in the number of detected change points. This explains the observed fluctuation in the precision rates.

The results for the recall rate and the ratio of change point numbers are presented in Figure A3 and Figure A4. Notably, the relatively high recall rate observed when *d* is equal to 2 and 10 may be misleading. Given the abundance of false change points identified under a small *d*, there is a higher probability that these false points are situated near the true change point. Thus, the elevated recall rate in the upper two panels does not accurately reflect the detection performance. The fluctuation trend in the ratio of change point numbers, as depicted in Figure A4, shows a similar fluctuation trend when *d* is 2 and 10, and the cause of this pattern is explained above. As *d* increases, the ratios of change point numbers experience a sharp decrease across all tested information criteria. However, the low precision and recall rates in the variance shift case indicate that utilizing information criteria penalized MLE for detecting the sole shift in variance may not be the optimal approach.

## Appendix B. Simulation and Discussion for Mean and Variance Change Case

In this section, we will provide a concise explanation of the scenario where both the mean and the variance of the time series change. This is a frequently encountered situation, as assuming a constant parameter in real-world scenarios is often a weak assumption. In general, varying combinations of changes in mean and variance magnitude will influence the overall detection effect. If the change magnitude in the mean significantly exceeds the change in variance, it can be considered a scenario of mean change with constant variance, and vice versa.

For a simple illustration, we adopt the same configuration used in the simulations for the mean change case. We specifically test the scenario with normally distributed time series data, where changes in both mean and variance are assumed to occur synchronously. Let the number of change points *K* increase from 1 to 20 with the parameter shifting pattern

$$\left(\mu ,\sigma^{2}\right)\overset{\tau_{1}}{⟶}\left(\mu +\Delta \mu ,\sigma^{2}+\Delta \sigma^{2}\right)\overset{\tau_{2}}{⟶}\left(\mu ,\sigma^{2}\right)\overset{\tau_{3}}{⟶}\ldots \overset{\tau_{19}}{⟶}\left(\mu +\Delta \mu ,\sigma^{2}+\Delta \sigma^{2}\right)\overset{\tau_{20}}{⟶}\left(\mu ,\sigma^{2}\right).$$

where the initial parameter setup $\left(\mu ,\sigma^{2}\right)=\left(1,1\right)$. The change magnitude Δμ=1.25 and $\Delta \sigma^{2}=8$ are selected. The precision rate, recall rate, and ratio of change point numbers under different d=2,10,25,or50 are given in Figure A5, Figure A6, Figure A7.

When the minimum segment distance *d* is small, the overall precision rate, recall rate, and ratio of change point numbers exhibit similarity to the results in the scenario of variance change solely, but with improvements across all three indices (due to the introduction of mean change). Furthermore, the overall detection performances improve with an increase in *d*. Nevertheless, even when *d* increases to 50, there is still a discernible gap in the detection performances compared to the scenario of mean change alone. In practical applications, when employing information criteria penalized MLE for change point detection, the judicious selection of the minimum distance between change points is a crucial consideration. This task may require expertise in the specific application field or the assistance of domain experts.
